# Impact of Real-Time Continuous Glucose Monitoring Data Sharing on Quality of Life and Health Outcomes in Adults with Type 1 Diabetes

**DOI:** 10.1089/dia.2020.0466

**Published:** 2021-02-25

**Authors:** William H. Polonsky, Addie L. Fortmann

**Affiliations:** ^1^ Behavioral Diabetes Institute, San Diego, California.; ^2^ Department of Medicine, University of California, San Diego, California.; ^3^ Scripps Whittier Diabetes Institute, Scripps Health, La Jolla, California, USA.

**Keywords:** Continuous glucose monitoring, Data sharing, Type 1 diabetes, Quality of life

## Abstract

***Background:*** To examine experiences with real-time continuous glucose monitoring (RT-CGM) data sharing and its impact on health-related outcomes.

***Methods:*** Adults with type 1 diabetes (T1D) (*N* = 302) using the Dexcom G5 Mobile or G6 RT-CGM system and sharing data with ≥1 family/friend follower completed a survey exploring their perceived value of data sharing, the impact of sharing on health and quality of life (QoL) outcomes, and how their chief follower (CF) used shared data to support their diabetes management. Regression analyses examined whether CF actions were linked to reported changes in health and QoL outcomes for the T1D adult.

***Results:*** The majority had lived with T1D >10 years, (76.5%), used RT-CGM >1 year (58.0%), and identified their spouse/partner as CF (51.9%). Data sharing reportedly contributed to improved hypoglycemic confidence (for 89.4% of respondents), improved overall well-being (54.3%), and reduced diabetes distress (36.1%). Benefits related to data sharing included fewer episodes of severe hypoglycemia (62.2%), better sleep (52.4%), and A1C improvement (47.3%). In particular, three positive CF actions were independent predictors of health and QoL benefits: celebrating success related to glycemic control, providing encouragement when glycemic control is challenging, and teamwork discussions about how CF should respond to out-of-range values.

***Conclusions:*** RT-CGM data sharing was associated with a range of health and QoL-related benefits. The occurrence of benefits was influenced by the collaborative management approaches taken by RT-CGM users and their data-sharing followers. Longitudinal trials are needed to determine the most effective patterns of collaborative data sharing, leading to their implementation into routine diabetes management.

## Introduction

Recent advances in real-time continuous glucose monitoring (RT-CGM) systems now provide the opportunity for remote data sharing within one's personal network (e.g., friends, family, and health care professionals). Once the RT-CGM user—most typically, an individual with type 1 diabetes (T1D)—grants access, the selected followers have the opportunity to view the user's real-time data on their smartphones or smart watches and, with certain systems, to be alerted if the user's glucose values reach certain thresholds (e.g., indicating severe hypoglycemia or hyperglycemia). Despite growing use of these features, little is known about data sharing practices or their potential impact on quality of life (QoL) and health outcomes for the T1D individual and his/her followers.

Recently, in a large retrospective study of youths with T1D who were using RT-CGM and had downloaded the necessary data sharing app, Welsh et al. found that glycemic outcomes were significantly better among those who had at least one active data-sharing follower versus those who had no followers.^1^ Although the direction of causality is uncertain, this may suggest a potential glycemic benefit due to data sharing. Early qualitative evidence suggests real-time sharing of RT-CGM data contributes to greater feelings of safety, both for the individual with T1D as well as their data sharing followers. However, also highlighted were interpersonal challenges related to its use.^2^ Similarly, a study involving semistructured interviews with 20 parents of T1D primary schoolers found that remote CGM data sharing was viewed mostly as beneficial, often contributing to improved sleep quality and reduced anxiety for the parents, although also pointing to the potential for family conflict.^3^

In a series of interviews with adult RT-CGM users and their spouses conducted before the advent of remote data sharing, Ritholz et al. described the mixed emotional and interpersonal impact of RT-CGM, highlighting greater positive comfort and collaboration between partners as well as, on occasion, greater conflict around personal boundaries concerning the true “ownership” data. Furthermore, decisions regarding RT-CGM user's versus partner's responsibility for taking needed action (especially when hypoglycemic events occur) were problematic.^4^ With remote sharing of RT-CGM data now available, it is possible that the emotional benefits may be greater, but that miscommunication and family conflict may also be enhanced. The paucity of studies to date makes it difficult to draw conclusions.

What exactly is the value of remote data sharing, and to what degree does it influence QoL and health-related outcomes? Furthermore, does the manner in which RT-CGM users and their followers respond to and communicate about the viewed data contribute to its utility? To investigate these issues, we surveyed three large cohorts of current users of remote real-time CGM data sharing: T1D adults, spouses/partners of T1D adults, and parents of T1D children. For each group, we explored data sharing experience, the reported reactions and behaviors in response to remote CGM data, the perceived value of remote data sharing, and the impact of data sharing on key aspects of QoL and health outcomes. This report focuses solely on the survey results from the cohort of T1D adults.

## Research Design and Methods

Adults who self-identified as having T1D were recruited from the Dexcom database through an e-mail invitation. Inclusion criteria were T1D for at least 12 months, age ≥25 years, currently using the Dexcom RT-CGM (either the Dexcom G5 Mobile or Dexcom G6 Systems), and Dexcom's data sharing app (“Share”), indicates that their most important or “chief” follower (CF) is a spouse/partner, adult family member, or close friend (i.e., not a health care professional), and has been sharing with that CF >3 months.

The e-mail invitation explained that the study involved the completion of an online questionnaire examining the feelings and experiences of people with T1D regarding the Dexcom data-sharing feature. Potential participants were informed that the study was being conducted in collaboration between Dexcom and the Behavioral Diabetes Institute, that survey responses were anonymous, and that participation was voluntary. Respondents were asked to access an online portal and complete eight screening items, and if eligible, an informed consent document and an ∼80-item questionnaire. Completers received a $25 electronic gift card for participation. All collected data were entered into a central database using a Health Insurance Portability and Accountability (HIPAA)-protected server, with no linkages to personal health information or personal identifiers. The research protocol was approved by Ethical and Independent Review Services, a community-based institutional review board.

### Measures

A co-design approach was taken. After a review of the current literature, we completed semi-structured interviews with five T1D adults who were currently using the Dexcom G5 or G6 RT-CGM systems and were actively sharing their real-time data with a spouse/partner, adult family member, or close friend. The interviews focused on attitudes toward RT-CGM data sharing, key elements of satisfaction and dissatisfaction with data sharing, the various ways in which the T1D adult and his/her CF communicated about, and responded to, the shared data, and the ways in which data sharing had influenced QoL and clinical outcomes. Based on these data, we developed a four-section self-report questionnaire battery in collaboration with our five interviewees.

Section 1 (“Sample Characteristics”) focused on respondents' demographic information (age, gender, ethnicity, years of education, and number of years since T1D diagnosis), personal experience with RT-CGM, and CF attributes. Section 2 (“CF's Use of RT-CGM Shared Data”) examined respondent's perspective on how their CF generally responded to/acted on the shared data (Table 2). Section 3 (“Perceived *Value* of RT-CGM Data Sharing”) assessed the respondent's perspectives on the value of RT-CGM data sharing with their CF (Table 3). For Sections 2 and 3, items were scored on a 5-point Likert scale (“strongly disagree,” “somewhat disagree,” “neutral,” “somewhat agree,” and “strongly agree”).

Finally, Section 4 (“Perceived *Impact* of RT-CGM Data Sharing”) examined respondents' perceptions of the impact of RT-CGM data sharing on key aspects of QoL and health outcomes. Since no existing validated instruments assess the individual's perception of change retrospectively, well-validated measures frequently used in previous RT-CGM studies were adapted for use in this study.

To evaluate change in feelings about hypoglycemia, the nine-item Hypoglycemic Confidence Scale was employed (HCS sample item: “feeling confident that I can stay safe from serious problems with hypoglycemia when exercising”).^5^ In the modified version, respondents were asked to indicate how data sharing had affected them on a 5-point Likert scale (“much less confident than before,” “somewhat less confident than before,” “neutral,” “somewhat more confident than before,” and “much more confident than before”).

To evaluate worries and concerns related to diabetes and its management, the 28-item Diabetes Distress Scale for Adults with T1D was included (T1-DDS sample item: “Feeling worried that I will develop serious long-term complications, no matter how hard I try”). In this similarly modified version, respondents indicated how data sharing had affected them on a 5-point scale (“much more of a problem than before,” “somewhat more of a problem than before,” “no change,” “somewhat less of a problem than before,” and “much less of a problem than before”). The T1-DDS has seven diabetes-specific subscales: powerlessness (feeling discouraged or helpless about one's own T1D), management distress (feeling disappointed with one's own self-care efforts), hypoglycemia distress (concerns about possible hypoglycemic events), negative social perceptions (feeling judged or stigmatized by others), physician distress (feeling disappointed with one's current health care professionals), eating distress (concerns that one's eating is out of control), and family/friends distress (feeling that there is too much focus on T1D among loved ones).^6^

Finally, to evaluate overall well-being, the World Health Organization-5 scale was used (WHO-5 sample item: feeling “cheerful and in good spirits”), with respondents again rating how data sharing had affected them on a similar 5-point scale (“much less of the time,” “somewhat less of the time,” “no change,” “somewhat more of the time,” or “much more of the time”).^7^

Regarding health outcomes, respondents rated how data sharing had affected their sleep quality (“getting ‘much more’ ‘somewhat more’, ‘no change’, ‘somewhat less', or ‘much less' quality sleep than I used to”), frequency of severe hypoglycemic episodes (“having ‘many fewer’ ‘somewhat fewer’, ‘no change’, ‘somewhat more’, or ‘many more’ severe episodes than I used to”) and glycemic control (“A1C has ‘dropped a lot’ ‘dropped a little’, ‘no change’, ‘risen a little’, or ‘risen a lot’ ”).

Throughout the questionnaire battery, respondents were frequently reminded that they were *not* being asked to evaluate the impact of RT-CGM overall, but only how one specific aspect of RT-CGM use—data sharing with the CF—had affected them.

### Data analysis

Descriptive statistics (*N*, %, mean, standard deviation) were used to describe T1D respondents' demographics and diabetes history, and their reported use of RT-CGM and data sharing. Frequencies, reported as *N* (%), were obtained to summarize the CF's use of shared RT-CGM data, the perceived value of RT-CGM data sharing, and the perceived impact of RT-CGM data sharing on QoL (HCS, T1-DDS, and WHO-5) and health outcomes (sleep quality, severe hypoglycemia frequency, and A1C), all from the perspective of the T1D respondent.

All analyses were item-level, with the exception of the (modified) QoL survey outcomes, which were calculated as item means. Mean scores for all QoL scales (as well as subscales, in the case of the T1-DDS) ranged from 1.0 to 5.0, with lower scores reflecting a relatively positive impact of Share on QoL, and higher scores indicating a relatively negative impact. As 3 (“no change”) was the neutral response option for all QoL *items*, a mean of 3.0 was also interpreted as the neutral/“no change” score on the QoL scale and subscales. QoL mean score thresholds were then selected to represent respondents' perception of Share's impact on their QoL: “Improved” (<2.5), “No Change” (≥2.5 and <3.5), and “Worsened” (≥3.5).

Finally, multiple linear regression analyses were conducted to evaluate whether the ways in which the CF used and responded to the RT-CGM data influenced important QoL and other health outcomes. Specifically, the six CF RT-CGM relevant skills and behaviors were entered as independent variables in separate multiple regressions for each of the three QoL outcomes (HCS, T1-DDS, and WHO-5) and each of the three health outcomes (sleep quality, severe hypoglycemia frequency, and A1C). Key demographic variables (age, gender, years since diagnosis and CF type) were included as covariates in each of the six regression models. IBM SPSS Statistics 25 was used for all analyses.

## Results

### Clinical characteristics of the sample

Of the 315 T1D adults who began the survey, 302 (95.9%) completed it satisfactorily. Respondents were predominantly non-Hispanic white (88.1%), female (59.6%), and well educated (58.3% college graduates). Mean age was 42.8 ± 12.8 years. The majority had been living with T1D >10 years (76.5%) and had been using CGM >1 year (58.0%). Although the majority of respondents (58.3%) reported having more than one follower, most (51.9%) indicated that their CF was their spouse/partner. Descriptive statistics are presented in Table 1.

**Table 1.** Sample Characteristics (*N* = 302)

	*N* (%)
Participant demographics	
Age, in years (mean ± SD)	42.8 ± 12.8
Female	180 (59.6%)
Non-Hispanic white	266 (88.1%)
College graduate	176 (58.3%)
Married or living with partner	252 (83.4%)
Duration of T1D	
≤5 years	50 (16.6%)
6–10 years	21 (7.0%)
>10 years	231 (76.5%)
Duration of CGM use	
3–6 Months	43 (14.2%)
7–12 Months	84 (27.8%)
>1 Year	175 (58.0%)
Number of followers	
*1*	126 (41.7%)
*2*	123 (40.7%)
*>2*	53 (17.6%)
Spouse/partner as CF	157 (51.9%)
Duration of sharing with CF	
<3 Months	2 (<1%)
3–6 Months	65 (21.5%)
7–12 Months	97 (32.1%)
>1 Year	138 (45.7%)

*N* (%) presented unless otherwise noted.

CF, chief follower; CGM, continuous glucose monitoring; SD, standard deviation; T1D, type 1 diabetes.

### CFs' use of RT-CGM shared data

More than half of respondents (53.6%) indicated that their CF typically checked their CGM readings multiple times/day, whereas other CFs reportedly checked less frequently—only several times/week (29.5%) or once weekly or less (16.9%). Most respondents (92.4%) agreed (“somewhat” or “strongly”) that the CF knew what to do when they observed glucose values in the hypoglycemic range (“Hypoglycemic Knowledge”) (Table 2). Furthermore, 69.5% agreed that they and their CF had discussed how best to respond (and how not to respond) when seeing out-of-range glucose values (“Clear Discussion”). Similar majorities agreed that their CF offered encouragement when noticing that they were struggling with glucose management (“Offered Encouragement,” 69.9%) and celebrated with them when they observed glucose management to be going well (“Celebrated,” 57.3%). However, drawbacks were apparent, with many respondents agreeing that the CF was now “bugging them too much” about glucose readings (“Bugs Me,” 17.5%) and did not know how best to respond to glucose data they were receiving (“Lack of Understanding,” 14.6%).

**Table 2.** Chief Followers Use of Shared Real-Time Continuous Glucose Monitoring Data

	Disagree (*n*/%)	Neutral (*n*/%)	Agree (*n*/%)
Celebrates: “When seeing my numbers, my CF celebrates with me when things are going well”^a^	45 (14.9)	84 (27.8)	173 (57.3)
Lack of Understanding: “My CF does not understand how best to respond when seeing my numbers”^a^	208 (68.9)	50 (16.5)	44 (14.6)
Offers Encouragement”: My CF offers the encouragement needed when I am struggling with my numbers”^a^	27 (8.9)	64 (21.2)	211 (69.9)
Hypoglycemic Knowledge”: “My CF knows just what to do if he/she sees that my BG is getting too low”^a^	13 (4.3)	10 (3.3)	279 (92.4)
Bugs Me: “Because of data sharing, my CF now bugs me too much about numbers”^a^	209 (69.2)	40 (13.2)	53 (17.6)
Clear Discussion: “We had had a clear discussion about how my CF should best respond (or not) when seeing that my numbers are out of range”^a^	38 (12.6)	54 (17.9)	210 (69.5)

Data reflect T1D respondent's perception of CF's use of shared RT-CGM data.

^a^ Items were rated on a 5-point Likert response scale: 5 = “strongly agree,” 4 = “somewhat agree,” 3 = “neutral,” 2 = “somewhat disagree,” 1 = “strongly disagree.” Categories 5 and 4 (“agree”), and 2 and 1 (“disagree”) were collapsed for presentation.

BG, blood glucose; RT-CGM, real-time continuous glucose monitoring.

### Perceived *value* of RT-CGM data sharing with CF

Respondents indicated a high level of overall satisfaction with RT-CGM data sharing, scoring 8.6 ± 1.5 on a 10-point scale. As detailed in Table 3, common benefits included greater peace of mind (89.1%) and feeling less alone with diabetes (79.5%). Many also felt that such sharing had contributed to a better relationship with their CF (41.4%). Furthermore, 79.5% reported that their CF were now more understanding about how challenging diabetes can be. Although negative perspectives were less common, 23.5% of participants reported feeling more judged by their CF than before, 9.3% felt that data sharing had led to more tension with their CF and 8.9% that it had given their follower too much information.

**Table 3.** Perceived Value of Real-Time Continuous Glucose Monitoring Data Sharing

	Disagree (*n*/%)	Neutral (*n*/%)	Agree (*n*/%)
Data sharing gives me peace of mind^a^	20 (6.6)	13 (4.3)	269 (89.1)
Data sharing gives my CF peace of mind^a^	5 (1.7)	13 (4.3)	284 (94.0)
Data sharing makes me feel less alone with my diabetes^a^	14 (4.6)	48 (15.9)	240 (79.5)
Diabetes sharing has improved the relationship with my CF^a^	18 (6.0)	159 (52.6)	125 (41.4)
Because of data sharing, my CF is more understanding about how challenging diabetes can be^a^	20 (6.6)	42 (13.9)	240 (79.5)
Because of data sharing, I feel more judged than before^a^	180 (59.6)	51 (16.9)	71 (23.5)
Data sharing has caused more tension in my relationship with my CF^a^	247 (81.8)	27 (8.9)	28 (9.3)
Data sharing has given my CF too much information^a^	243 (80.5)	32 (10.6)	27 (8.9)

*N* (%) presented unless otherwise noted.

^a^ Items rated on a 5-point Likert response scale: 5 = “strongly agree,” 4 = “somewhat agree,” 3 = “neutral,” 2 = “somewhat disagree,” 1 = “strongly disagree.” Categories 5 and 4 (“agree”), and 2 and 1 (“disagree”) were collapsed for presentation.

### Perceived *impact* of RT-CGM data sharing on QoL and health outcomes

On the modified HCS, 89.4% of respondents reported that data sharing had helped them to become more confident in their ability to avoid or manage hypoglycemia (Table 4). Similarly, on the WHO-5, 54.3% indicated that data sharing had contributed to greater overall well-being. On the modified T1-DDS, 36.1% indicated diabetes distress had fallen due to data sharing, although the majority reported no change. Of the seven T1-DDS subscales, the greatest benefits were seen in hypoglycemia distress (60.6% reported improvement) and management distress (42.7% reported improvement). The most notable drawback was in the friends/family distress subscale, where 20.5% of respondents indicating that data sharing had enhanced distress in this area.

**Table 4.** Perceived Impact of Real-Time Continuous Glucose Monitoring Data Sharing on Quality of Life and Health Outcomes

	*N* (%) improved	*N* (%) no change	*N* (%) worsened
QoL outcome^a^			
HCS	270 (89.4)	31 (10.3)	1 (0.3)
WHO-5 Well-Being Scale	164 (54.3)	134 (44.4)	4 (1.3)
Diabetes Distress Scale (T1-DDS)			
Overall	109 (36.1)	189 (62.6)	4 (1.3)
Powerlessness	85 (28.1)	175 (57.9)	42 (13.9)
Management distress	129 (42.7)	159 (52.6)	14 (4.6)
Hypoglycemia distress	183 (60.6)	106 (35.1)	13 (4.3)
Negative social perceptions	79 (26.2)	211 (69.9)	12 (4.0)
Eating distress	104 (34.4)	173 (57.3)	25 (8.3)
Physician distress	98 (32.5)	184 (60.9)	20 (6.6)
Friend/family distress	85 (28.1)	155 (51.3)	62 (20.5)
Health outcome^b^			
HbA1c^c^	143 (47.4)	101 (33.4)	6 (2.0)
Severe hypoglycemic events^d^	188 (62.3)	111 (36.7)	3 (1.0)
Sleep quality^d^	158 (52.3)	118 (39.1)	26 (8.6)

^a^ For table presentation purposes, QoL mean score thresholds were then selected to represent respondents' perception of Share's impact on their QoL: “Improved” (1.0–2.4), “No Change” (2.5–3.4), and “Worsened” (3.5–5.0).

^b^ For table presentation purposes health item response options were collapsed as follows: 1 and 2 = “Improved”; 3 = “No Change”; 4 and 5 = “Worsened.”

^c^ Participants used a 5-point Likert scale to indicate the impact of Share on their HbA1c: 1 = “has dropped a lot (at least 0.5%),” 2 = “has dropped a little (but <0.5%),” 3 = “has not really changed, 4 = “has risen a little (but <0.5%),” 5 = “has risen a lot.” Note, percentages do not sum to 100% as *n* = 52 (17.2%) reported being “unsure” of the impact on HbA1c and were excluded from these counts.

^d^ Participants reported the perceived impact of share on the frequency of severe hypoglycemic events and amount of quality sleep (respectively) on a 5-point Likert scale: 1 = “many fewer/much more than I used to,” 2 = “somewhat fewer/more than I used to,” 3 = “no change,” 4 = “somewhat more than I used to,” 5 = “many/much more than I used to.”

DDS-T1D, Diabetes Distress Scale for type 1 diabetes; HbA1c, glycosylated hemoglobin; HCS, hypoglycemia confidence scale; QoL, quality of life; WHO, World Health Organization.

Health benefits in response to data sharing included 52.4% of participants noting (“somewhat” or “much”) higher quality sleep, 62.2% reporting (“somewhat” or “much”) fewer episodes of severe hypoglycemia, and 47.3% indicating A1C improvement.

### Are CF behaviors associated with the perceived changes in QoL and clinical outcomes?

No consistent associations were observed between any of the demographic covariates (age, gender, years since diagnosis, and CF type) and the QoL (HCS, T1-DDS, and WHO-5) or health outcomes (sleep quality, severe hypoglycemia frequency, and A1C). However, with control for these demographics, three of the CF skills/behaviors emerged as significant independent predictors of the QoL and health outcomes in a relatively consistent pattern (Tables 5 and 6).

**Table 5.** Associations of Chief Followers Use of Shared Real-Time Continuous Glucose Monitoring Data with Quality of Life Outcomes

	HCS^a^	WHO-5^a^	DDS*^b^*overall	DDS powerlessness^b^	DDS management^b^	DDS hypoglycemia^b^	DDS negative social perceptions^b^	DDS eating^b^	DDS physician distress^b^	DDS family/friends^b^
Covariates	β	β	β	β	β	β	β	β	β	β
Age	<0.01	0.02	−0.04	−0.11^*^	<0.01	−0.04	<0.01	−0.05	0.04	−0.03
Gender (1 = female, 2 = male)	<0.01	<0.01	0.09	0.14^**^	0.06	0.01	0.09	0.10	0.09	<0.01
T1D duration	−0.03	−0.13^*^	0.05	0.05	<0.01	0.06	0.08	0.07	0.06	−0.04
Spouse/partner as CF (1 = yes, 0 = no)	−0.02	−0.01	0.01	0.01	0.03	0.02	0.02	−0.05	0.04	−0.02
CF use of shared RT-CGM data^c^										
Celebrates: “When seeing my numbers, my CF celebrates with me when things are going well”	0.17^**^	0.25^***^	−0.17^*^	−0.10	−0.15^*^	−0.17^*^	−0.19^**^	−0.12	−0.15^*^	−0.10
Lack of understanding: “My CF does not understand how best to respond when seeing my numbers”	−0.11	−0.04	0.13^*^	0.12^*^	0.09	0.13^*^	0.07	0.06	0.08	0.16^**^
Offers encouragement”:My CF offers the encouragement needed when I am struggling with my numbers”	0.13	0.07	−0.14^*^	−0.12	−0.20^**^	−0.07	−0.15^*^	−0.10	−0.10	−0.09
Hypoglycemic knowledge”: “My CF knows just what to do if he/she sees that my BG is getting too low”	0.13^*^	0.05	−0.04	−0.05	−0.03	−0.06	0.02	−0.03	−0.05	−0.04
Bugs me: “Because of data sharing, my CF now bugs me too much about numbers”	0.05	0.02	0.08	0.08	0.06	−0.03	0.01	0.07	0.02	0.23^***^
Clear discussion: “We had had a clear discussion about how my CF should best respond (or not) when seeing that my numbers are out of range”	0.18^**^	0.14^*^	−0.15^**^	−0.15^**^	−0.09	−0.16^**^	−0.11	−0.13^*^	−0.10	−0.11

^*^ *P* < 0.05; ^**^P < 0.01; ^***^ *P* < 0.001.

^a^ Higher scores reflect *higher* QOL.

^b^ Higher scores reflect *lower* QOL.

^c^ Items were rated on a 5-point Likert response scale (5 = “strongly agree,” 4 = “somewhat agree,” 3 = “neutral,” 2 = “somewhat disagree,” and 1 = “strongly disagree”).

**Table 6.** Associations of Chief Followers Use of Shared Real-Time Continuous Glucose Monitoring Data with Clinical Outcomes

	HbA1c*^a^*(*β*)	Severe hypoglycemia*^b^*(*β*)	Sleep*^c^*(*β*)
Covariates			
Age	0.06	−0.12^*^	0.03
Gender (1 = female, 2 = male)	−0.01	< 0.01	−0.06
T1D duration	−0.09	−0.09	0.05
Spouse/partner as CF (1 = yes, 0 = no)	<0.01	−0.09	−0.07
CF use of shared RT-CGM data^d^			
Celebrates: “When seeing my numbers, my CF celebrates with me when things are going well”	−0.03	−0.14^*^	−0.23^**^
Lack of understanding: “My CF does not understand how best to respond when seeing my numbers”	−0.08	0.02	0.03
Offers encouragement: My CF offers the encouragement needed when I am struggling with my numbers”	−0.08	−0.16^*^	0.08
Hypoglycemic knowledge: “My CF knows just what to do if he/she sees that my BG is getting too low”	−0.11	−0.10	−0.05
Bugs me: “Because of data sharing, my CF now bugs me too much about numbers”	0.10	−0.06	0.02
Clear discussion: “We had had a clear discussion about how my CF should best respond (or not) when seeing that my numbers are out of range”	−0.06	−0.01	−0.12^*^

^*^ *P* < 0.05. ^**^P < 0.01. ^***^P < 0.001.

^a^ Higher scores reflect *poorer* glycemic control. Items assessed the impact of Share on HbA1c, and was rated on a 5-point Likert response scale: 1 = “My HbA1c has dropped a lot (at least 0.5% or more),” 2 = “My HbA1c has dropped a little (but <0.5%),” 3 = “My HbA1c has not really changed,” 4 = “My HbA1c has risen a little (but <0.5%),” and 5 = “My HbA1c has risen a lot (at least 0.5% or more).” The *n* = 52 who responded “I'm not sure” were excluded from the HbA1c outcome analyses only.

^b^ Higher scores reflect *more frequent* severe hypoglycemia. Items assessed the impact of Share on severe hypoglycemic episode frequency on a 5-point Likert scale: 1 = “many fewer,” 2 = “somewhat fewer,” 3 = “no change,” 4 = “somewhat more,” 5 = “many more.”

^c^ Higher scores reflect *worse* sleep quality. Item assessed the impact of Share on the amount of quality sleep participants have, and was rated on a 5-point Likert response scale: 1 = “much more,” 2 = “somewhat more,” 3 = “no change,” 4 = “somewhat less,” 5 = “much less.”

^d^ Items were rated on a 5-point Likert response scale: 5 = “strongly agree,” 4 = “somewhat agree,” 3 = “neutral,” 2 = “somewhat disagree,” 1 = “strongly disagree.”

First, “Celebrated” was independently associated with data sharing-related improvements in hypoglycemic confidence (HCS, *P* < 0.01), overall well-being (WHO-5, *P* < 0.001), diabetes distress (T1-DDS, *P* < 0.05), sleep quality (*P* < 0.001), and severe hypoglycemia (*P* < 0.05). Second, a similar pattern was seen with “Clear Discussion,” with this variable being independently linked with improvements in hypoglycemic confidence (HCS, *P* < 0.01), overall well-being (WHO-5, *P* < 0.05), diabetes distress (T1-DDS, *P* < 0.001), and sleep quality (*P* < 0.05). Third, “Offers Encouragement” was independently associated with data sharing-related drops in diabetes distress (T1-DDS, *P* < 0.05) and severe hypoglycemia (*P* < 0.05). Finally, worsening in the family/friends distress dimension of the T1-DDS was independently linked with two of the reported CF behaviors, “Bugs Me” (*P* < 0.001) and “Lack of Understanding” (*P* < 0.01).

## Discussion

Consistent with the earlier findings of Litchman et al.,^2^ our results point to broad QoL and health benefits associated with data sharing in this large sample of adult RT-CGM users currently sharing their real-time data with at least one friend or family member. Approximately half or more of respondents indicated fewer episodes of severe hypoglycemia (62.2%), better sleep (52.4%) and A1C improvement (47.3%) due to data sharing, along with improvement in overall well-being (54.3%) and reduced diabetes distress (36.1%). Of note, the largest and most pervasive affective changes were linked to hypoglycemic concerns—89.4% of respondents indicated improvements in hypoglycemic confidence, whereas similar majorities agreed that they now had greater peace of mind (89.1%) and that data sharing had helped them to feel less alone with their diabetes (79.5%).

In explanation of these reported QoL and health benefits, patterns of associations indicating three positive CF actions as key and independent predictors were identified. These included when the CF reportedly celebrated with the RT-CGM user after seeing that things were going well with his/her glucose values (linked to improvement in HCS, WHO-5, T1-DDS, sleep quality, and severe hypoglycemia frequency), when the CF offered encouragement when seeing that the RT-CGM user was struggling with his/her glucose readings (linked to improvement in DDS and severe hypoglycemia frequency), and when the CF and RT-CGM user had discussed clearly how it would be best for the CF to respond to out-of-range values (linked to improvement in HCS, WHO-5, T1-DDS, and sleep quality). Of interest, the majority of respondents in this study sample agreed that their CFs had engaged in each of these actions (Table 2).

A supportive teamwork approach to diabetes management has long been associated with improved biomedical outcomes, improved QoL, and reduced family conflict.^8^ These results indicate that many participants in this study experienced such a teamwork approach in concert with data sharing and that this was a key contributor to the reported health and QOL outcomes.

Despite few participants reporting that QoL or health outcomes had worsened, it was apparent that negative aspects of data sharing exist and that these should be examined further. Specifically, 23.5% noted that they now felt more judged by their CF, whereas 20.5% indicated a rise in the interpersonal aspects of diabetes distress (the friend/family subscale of the T1-DDS). The latter was linked to the two negative CF behaviors—the CF now bugging the RT-CGM user too much about their glucose readings and the CF not knowing how best to respond to glucose values. However, as predictors of the major QOL and health outcomes, the overall findings point to the more potent contribution of the positive CF behaviors (or the absence of those behaviors), not the negative CF behaviors.

In total, these findings align with the clinical experience of the research team with RT-CGM users and their families. T1D adults seem to benefit most from RT-CGM data sharing when they and their followers (typically, the spouse or partner) are operating together as a team. From the RT-CGM user's perspective, this means that his/her followers are seen as providing kudos and appropriate support, while remaining respectful of personal boundaries. When this does not occur, followers may be seen as spies, judges, or people who are trying to control the actions of the RT-CGM user. These actions are unlikely to be beneficial and may lead the RT-CGM user to disconnect from data sharing.^9^ As demonstrated in our study findings, the potential value of an open conversation between the interested parties at the outset of data sharing to set boundaries and agree on roles is advised.

There are several limitations of this study. First, although the sample was relatively large, it may not be representative of the broader population of T1D adults who are sharing their RT-CGM data. Only users of Dexcom's data sharing system were surveyed, so we cannot know for certain whether similar beneficial outcomes might be observed among users of other RT-CGM data sharing systems. Also, younger RT-CGM users (children, teens, and young adults <25 years) were not surveyed and it seems possible, and perhaps likely, that their use of data sharing and their feelings about its use (especially with parents) may be quite different than what is observed in the current findings.

Importantly, in the adult population that the study targeted, we do not know what selection biases might have contributed to why some RT-CGM users chose to respond to the study invitation and others did not. Individuals with more negative experiences may have been less willing to participate in this study or they may have discontinued data sharing and, therefore, have been ineligible to participate. Although these study findings may represent a more enthusiastic group of RT-CGM users of data sharing, we suspect they reflect what is possible when data sharing is done well. Lessons learned from these positive experiences could inform RT-CGM onboarding practices as well as future studies to determine best practice recommendations.

Furthermore, these data are cross-sectional only, and the three QoL-related measures were modified for this study to facilitate retrospective enquiry into changes due to data sharing. Future development and validation of these modified measures will be necessary. It should be acknowledged that all data regarding the knowledge, attitudes, and responses of the CFs are similarly drawn solely from respondents' perspectives and memories. Also, although respondents were frequently reminded that they were being asked to consider how data sharing only has contributed to outcomes (not the combined influence of data sharing with RT-CGM), it is possible that some participants may have unknowingly conflated these factors. In an attempt to address this issue, we compared the reported changes in QoL and health outcomes between respondents who had experience with RT-CGM before data sharing versus those who had started RT-CGM and data sharing simultaneously. There were no significant differences in outcomes between the two groups (data not shown).

In conclusion, RT-CGM data sharing was associated with a range of reported health and QoL-related benefits for the RT-CGM user. The occurrence of these benefits was influenced by the ways in which RT-CGM users and their data-sharing followers respond to those data. In particular, the positive actions of taken (or not taken) by the CF in reaction to real-time data may be most critical. Longitudinal trials are needed to better understand the impact of data sharing on health and Qol outcomes. In the meantime, it will be worthwhile to provide education regarding data sharing “etiquette” and, most importantly, the benefits of positive CF behaviors.

## References

[1] Welsh JB, Derdzinski M, Parker AS, et al.: Real-time sharing and following of continuous glucose monitoring data in youth. Diabetes Ther 2019;10:751–755 30701468 10.1007/s13300-019-0571-0 PMC6437306

[2] Litchman ML, Allen NA, Colicchio VD, et al.: A qualitative analysis of real-time continuous glucose monitoring data sharing with care partners: to share or not to share? Diabetes Technol Ther 2018;20:25–31 29154685 10.1089/dia.2017.0285

[3] Burckhardt MA, Fried L, Bebbington K, et al.: Use of remote monitoring with continuous glucose monitoring in young children with type 1 diabetes: the parents' perspective. Diabet Med 2019;36:1453–1459 31257642 10.1111/dme.14061

[4] Ritholz MD, Beste M, Edwards SS, et al.: Impact of continuous glucose monitoring on diabetes management and marital relationships of adults with Type 1 diabetes and their spouses: a qualitative study. Diabet Med 2019;31:47–54 10.1111/dme.12276 23819557

[5] Polonsky WH, Fisher L, Hessler D, Edelman SV: Investigating hypoglycemic confidence in type 1 and type 2 diabetes. Diabetes Technol Ther 2017;19:131–136 27997217 10.1089/dia.2016.0366

[6] Fisher L, Polonsky WH, Hessler DM, et al.: Understanding the sources of diabetes distress in adults with type 1 diabetes. J Diabetes Complications 2015;29:572–577 25765489 10.1016/j.jdiacomp.2015.01.012 PMC4414881

[7] Hajos TR, Pouwer F, Skovlund SE, et al.: Psychometric and screening properties of the WHO-5 well-being index in adult outpatients with type 1 or type 2 diabetes mellitus. Diabet Med 2013;30:63–69 10.1111/dme.12040 23072401

[8] Wiebe DJ, Helgeson V, Berg CA: The social context of managing diabetes across the life span. Am Psychol 2016;71:526–538 27690482 10.1037/a0040355 PMC5094275

[9] Messer LH, Johnson R, Driscoll KA, Jones J: Best friend or spy: a qualitative meta-synthesis on the impact of continuous glucose monitoring on life with type 1 diabetes. Diabet Med 2018;35:409–418 29247556 10.1111/dme.13568

